# Non-invasive detection of language-related prefrontal high gamma band activity with beamforming MEG

**DOI:** 10.1038/s41598-017-14452-3

**Published:** 2017-10-27

**Authors:** Hiroaki Hashimoto, Yuka Hasegawa, Toshihiko Araki, Hisato Sugata, Takufumi Yanagisawa, Shiro Yorifuji, Masayuki Hirata

**Affiliations:** 1 Osaka University, Endowed Research Department of Clinical Neuroengineering, Global Center for Medical Engineering and Informatics, Suita, 565-0871 Japan; 2 Osaka University Graduate School of Medicine, Department of Neurosurgery, Suita, 565-0871 Japan; 3 Osaka University Graduate School of Medicine, Division of Functional Diagnostic Science, Suita, 565-0871 Japan; 4 Osaka University Hospital, Department of Medical Technology, Suita, 565-0871 Japan; 5 Oita University, Faculty of Welfare and Health Science, Yufu, 879-5503 Japan; 6 National Institute of Information and Communications Technology, and Osaka University, Center for Information and Neural Networks (CiNet), Suita, 565-0871 Japan

## Abstract

High gamma band (>50 Hz) activity is a key oscillatory phenomenon of brain activation. However, there has not been a non-invasive method established to detect language-related high gamma band activity. We used a 160-channel whole-head magnetoencephalography (MEG) system equipped with superconducting quantum interference device (SQUID) gradiometers to non-invasively investigate neuromagnetic activities during silent reading and verb generation tasks in 15 healthy participants. Individual data were divided into alpha (8–13 Hz), beta (13–25 Hz), low gamma (25–50 Hz), and high gamma (50–100 Hz) bands and analysed with the beamformer method. The time window was consecutively moved. Group analysis was performed to delineate common areas of brain activation. In the verb generation task, transient power increases in the high gamma band appeared in the left middle frontal gyrus (MFG) at the 550–750 ms post-stimulus window. We set a virtual sensor on the left MFG for time-frequency analysis, and high gamma event-related synchronization (ERS) induced by a verb generation task was demonstrated at 650 ms. In contrast, ERS in the high gamma band was not detected in the silent reading task. Thus, our study successfully non-invasively measured language-related prefrontal high gamma band activity.

## Introduction

The language-related neural process is involved in higher processing and is a multifaceted and comprehensive brain function. Previously, we demonstrated the relationship between frequency bands and the location involved in language-related neural processing using electrocorticograms (ECoGs) and magnetoencephalography (MEG). First, using MEG, we revealed that a silent reading task induced event-related desynchronizations (ERDs) in the low gamma band (25–50 Hz) in the left prefrontal area and demonstrated that the laterality of the low gamma ERDs effectively predicted language dominance^1,2^. Second, we revealed that oscillatory changes ranging from the theta band (3–8 Hz) to the low gamma band were involved in neural process related to language function, depending on the band frequency^3^. The previous MEG study of Lam *et al*. also reported that the theta, alpha, beta, and gamma bands were all involved in sentence processing^4^.

The high gamma band (>50 Hz) activities recorded by ECoGs were reported to better reflect functional localization than the lower band^5^ and should therefore be a more reliable marker of cortical activation^6,7^. Previous studies using ECoGs have reported that high gamma band activities are involved in language and a language-related memory process using picture naming tasks or lexical decision tasks^8,9^. Oscillatory activities in the high-frequency band can be effectively measured using ECoGs, but they require invasive procedures to detect electrical activity on the cortical surface. In contrast, MEG is a non-invasive examination; however, its sensitivity in the high-frequency band is inferior to that of ECoGs. Therefore, previously, measuring language-related high gamma activities by MEG was believed to be difficult. In the present study, we aimed to detect language-related gamma band activities recorded with a 160-channel whole-head MEG system equipped with superconducting quantum interference device (SQUID) gradiometers using group statistical analyses and time-frequency analyses of beamformed MEG data. We used a verb generation task and a silent reading task because we have elucidated neuromagnetic oscillatory changes using these tasks previously^1–3,10–13^. Our final goal was a clinical application of language-related high gamma activities measured by MEG as an examination to determine the dominant hemisphere. If MEG was able to detect individual language-related high gamma activities robustly, non-invasive functional language mapping would be realized with higher spatial resolution than lower bands.

## Results

### High gamma band activities

**Figure 1:**
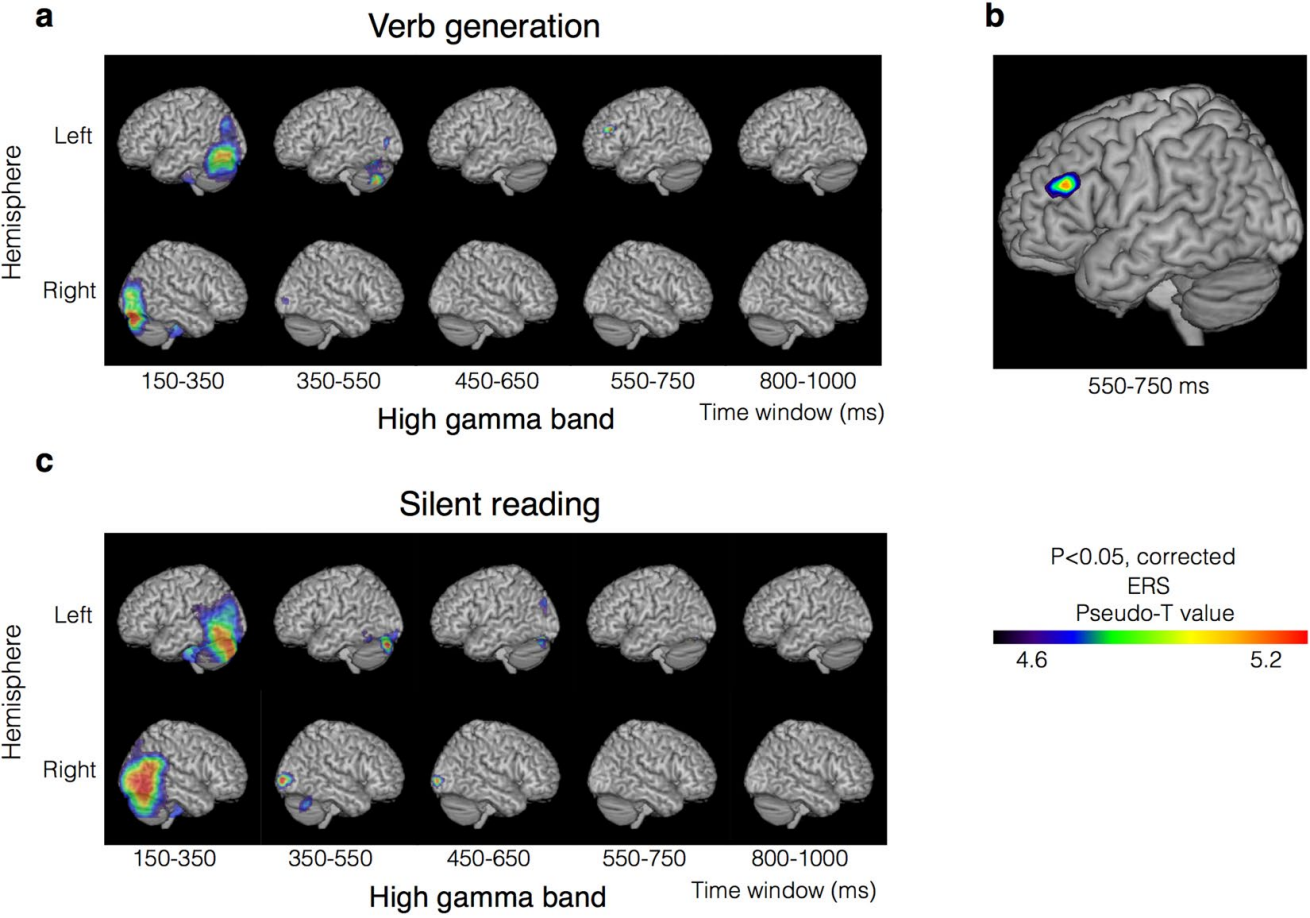
Distribution of high gamma ERSs. Group statistical images of beamformed analysis with a sliding time window with an overlap of 150 ms. (**a**) Statistically significant ERSs induced by the verb generation task are displayed. (**b**) Statistically significant ERSs induced by the verb generation task in the left MFG are displayed at 550–750 ms. High gamma ERSs appeared only in this time window. (**c**) Statistically significant ERSs induced by the silent reading task are displayed. Higher ERS power is indicated in red.

In the verb generation task, the transient power increased in the high gamma band that appeared in the left middle frontal gyrus (MFG; Brodmann area: BA 46) at the 550–750 ms post-stimulus window (corrected *p* = 0.0493; Fig. 1a,b), as revealed by beamformer group statistical analyses. Simultaneously, a silent reading task caused no high gamma event-related synchronization (ERS) in BA 46 (Fig. 1c). However, ERSs in the bilateral occipital cortices (BA 19) at 150–350 ms were obtained in both a verb generation task (corrected *p* = 0.006) and a silent reading task (corrected *p* = 0.006; Fig. 1a,c).

**Figure 2:**
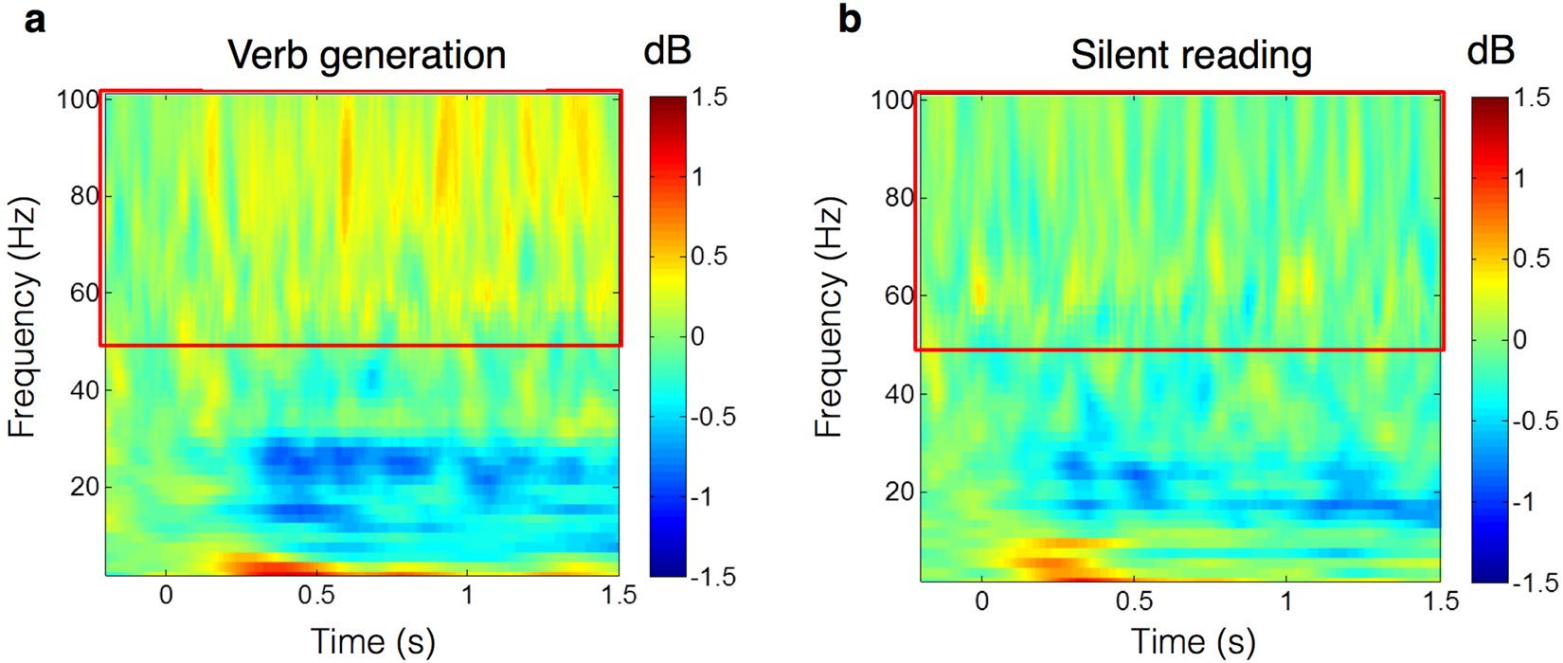
Grand averaged time-frequency spectrograms calculated by a virtual sensor. The area surrounded by the red frame corresponds to high gamma band frequencies. In the coloured bars, blue indicates ERDs, and red indicates ERSs. (**a**) In the verb generation task, the ERSs in the high gamma band appeared temporarily at approximately 650 ms. (**b**) In contrast, no ERSs were detected in the high gamma band during the silent reading task.

The coordinates at which the ERSs in the high gamma band were localized to the left MFG (BA 46) in the verb generation task were set as the target location of the virtual sensor for the time-frequency analysis. The target Montreal Neurological Institute (MNI) coordinates were [x, y, z] = [−54, 34, 26]. The time-frequency spectrograms demonstrated a dynamic change in brain activity. In the verb generation task, ERSs in the high gamma band appeared transiently at 650 ms and 950 ms. In contrast, a wide frequency range of ERDs from 300 to 1,000 ms appeared in the beta band (Fig. 2a). In the silent reading task, no ERSs were detected in the high gamma band (Fig. 2b). In the verb generation task, the 650 ms ERSs in the high gamma band obtained from the time-frequency analyses were concordant with those obtained from group statistical analyses. We compared the high gamma band power at 650 ms and 950ms between the verb generation task and the silent reading task. The results showed that the verb generation task induced significantly higher high gamma ERSs in the left MFG than the silent reading task at 650 ms (*p* = 0.0003 by paired t-test), but there were no significant differences at 950 ms (*p* = 0.288 by paired t-test).

**Figure 3:**
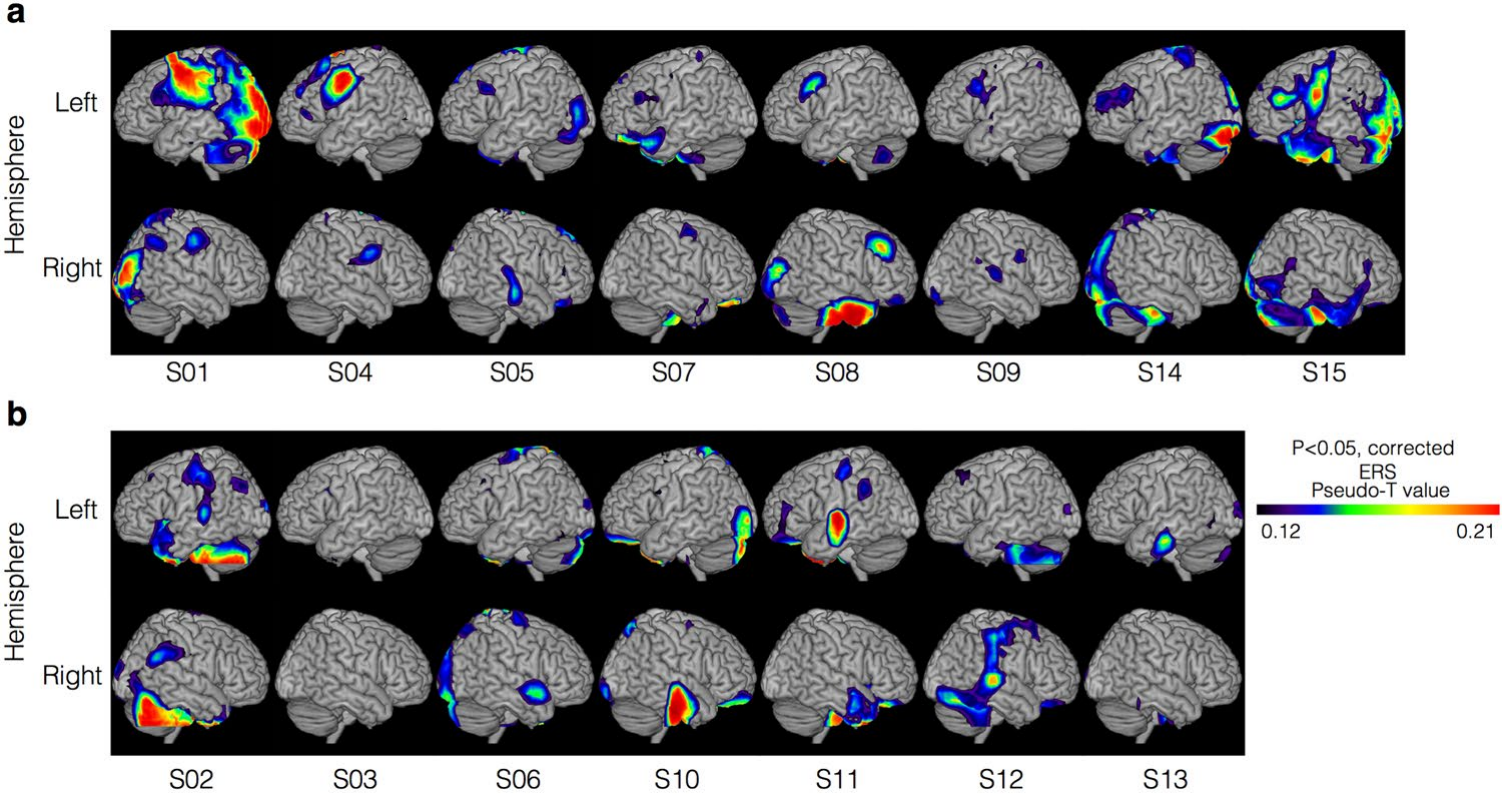
Individual beamformer results. High gamma ERSs induced by the verb generation task were detected in eight of the fifteen participants using the beamformer method. (**a**) High gamma ERSs were detected in the left MFG (BA 46) at 550–750 ms. (**b**) No high gamma ERSs were detected in the left BA 46 at 550–750 ms.

For each participant, high gamma ERSs (pseudo t value > 0.1) showed local maximum values in BA 46 by the verb generation task and detected with the beamformer method in eight of the fifteen participants (Fig. 3a,b). These results were not statistically significant.

### Alpha, beta and low gamma band activities

**Figure 4:**
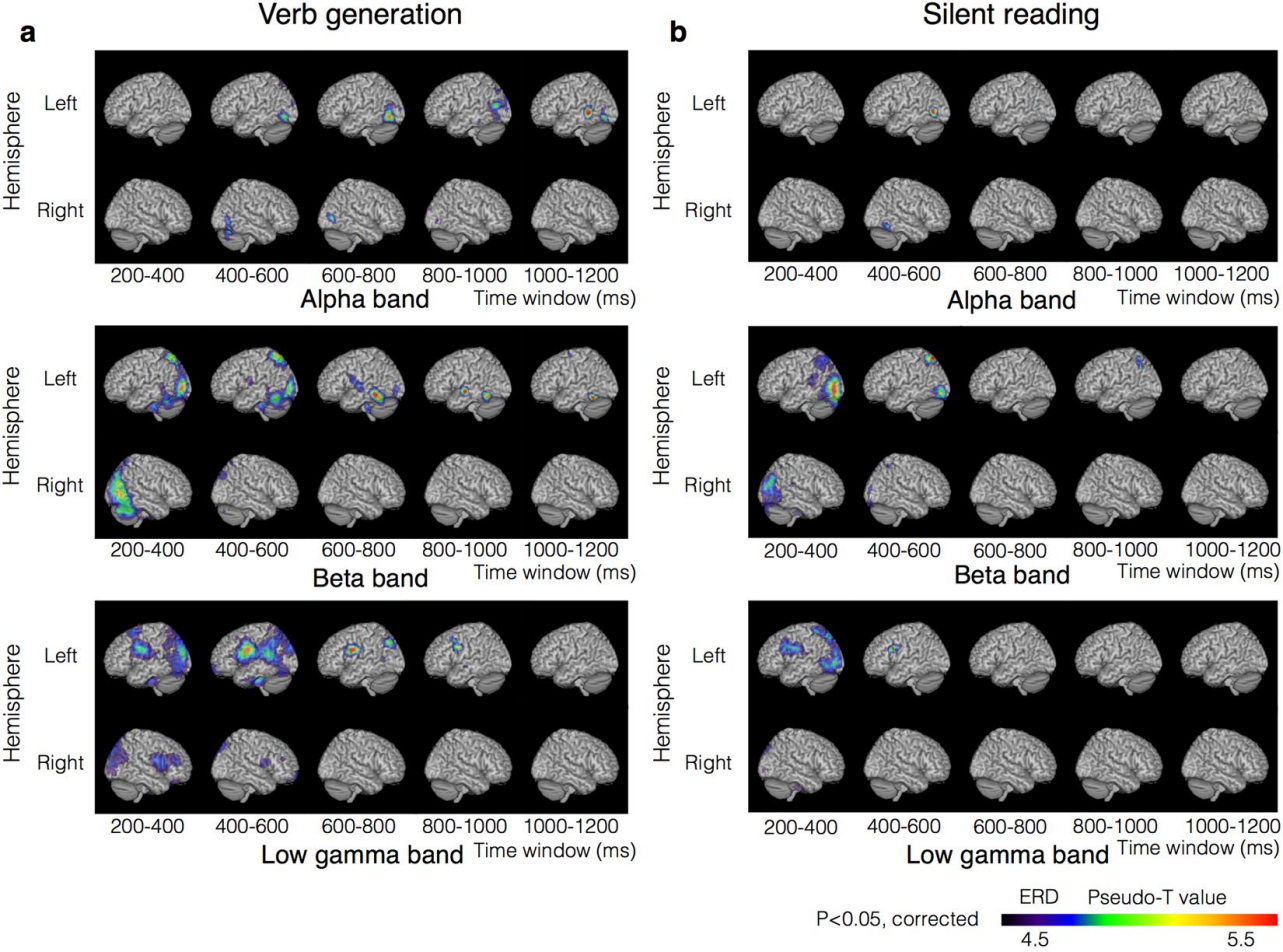
Distribution maps of sequential ERDs. Group statistical images of beamformed analysis with a sliding time window without an overlap. (**a**) Statistically significant ERDs induced by the verb generation task are displayed. (**b**) Statistically significant ERDs induced by the silent reading task are displayed. Higher ERD power is indicated in red.

In the verb generation task, sustained ERDs were observed in the alpha, beta, and low gamma bands (Fig. 4a). The ERDs in the alpha band appeared in the left middle temporal gyrus (MTG; BA 21) at 1,000–1,200 ms (corrected *p* = 0.012). In the beta band, the ERDs appeared serially in the left superior parietal lobule (SPL; BA 7) at 200–400 ms (corrected *p* = 0.006), the left inferior temporal gyrus (ITG; BA 20) at 600–800 ms (corrected *p* = 0.006) and the left MTG (BA 21) at 800–1,000 ms (corrected *p* = 0.0468). The ERDs in the low gamma band appeared in the left inferior frontal gyrus (IFG; BA 9) at 200–400 ms (corrected *p* = 0.006), 400–600 ms (corrected *p* = 0.006) and 600–800 ms (corrected *p* = 0.006).

In the silent reading task, ERDs in the bilateral occipital cortices (BA 19) appeared at 200–400 ms in the beta (corrected *p* = 0.006) and low gamma bands (corrected *p* = 0.006) and were similar to those obtained in the verb generation task. In the alpha band, ERDs appeared in the left MTG (BA 37) at 400–600 ms (corrected *p* = 0.0234). In the beta band, ERDs in the left SPL (BA 7) appeared at 200–400 ms (corrected *p* = 0.006) and 800–1,000 ms (corrected *p* = 0.0294). In the low gamma band, the ERDs in the left SPL (BA 7) appeared at 200–400 ms (corrected *p* = 0.006), as did the ERDs in the left IFG (BA 9) (corrected *p* = 0.006; Fig. 4b).

## Discussion

In this study, we non-invasively explored the frequency-dependent spatiotemporal distribution of neural activities involved in language processing using a beamformer method with a sliding time window and group analysis. We examined language-related high gamma ERSs non-invasively using MEG. In previous invasive studies using ECoGs, language-related high gamma ERSs were observed in the left frontal lobe^8,14,15^, but detecting language-related high gamma activities non-invasively by MEG was thought to be difficult. Only a very recent paper by Lam *et al*. has referred to high gamma activities associated with sentence processing in the left frontal and temporal regions using MEG^4^. However, the result was not statistically significant by multiple comparison. Therefore, the authors mainly focused on and discussed lower-frequency bands rather than the high gamma band. In our study, the low gamma ERDs observed in the left IFG during the silent reading task were consistent with our previous findings^1–3^. Similarly, the spatial distributions of ERDs in the alpha and beta bands were also consistent with our previous reports^2,3^. Additionally, for the verb generation task, the low gamma ERDs in the left prefrontal area were generally in agreement with our previous results^10^. Therefore, we believe that the findings of the present study are reasonable.

The role of high gamma band activities has not been fully described. However, many previous studies using ECoGs have reported that high gamma activities are widely involved in neural activities, including sensory^16^ and motor functions^5,17^, attention^18^, language^14,15^ and working memory^19^. Movement induces high gamma activities and a power decrease in the beta band^17^, and somatosensory stimulation induces high gamma activities and a power decrease in the alpha band^20^. In the present study, verb generation induced high gamma activities in tandem with decreased power in the low gamma band. Several studies have shown that high gamma activities reflect the firing of pyramidal neurons^21,22^ and that high gamma power is modulated by lower-frequency bands^23,24^. Therefore, high gamma activities are generally more spatiotemporally focal than lower-frequency activity and more accurately reflect neural function^5,17,23,25,26^. Although cortical stimulation mapping is accepted as the gold standard for language mapping, the high gamma activities recorded by ECoG are a reliable marker of cortical activation and enable functional language mapping with high spatial-temporal resolution^5,6,8,15,27^. High gamma band activities can be stably detected using ECoGs but are difficult to detect using electroencephalograms (EEGs) because high-frequency activities are both weak and attenuated by the skull bone; thus, they are too weak to be measured by skin electrodes^9^. Similarly, high gamma activities are difficult to detect using MEG because they are weak and attenuated proportionally to the square of the distance from the cortical activity. High gamma activities related to somatosensory^20^ or motor processing^28,29^ have been reported using MEG, but previously, measuring language-related high gamma activities by MEG was believed to be difficult. There are two possible reasons explaining why language-related high gamma activity was detected in the present study. First, we used a 160-channel whole-head MEG system with rather densely positioned sensors, which may have increased the possibility of detecting subtle high-frequency activity immediately under the sensors. Second, the verb generation task we used might have imposed a high task demand on the prefrontal language area^15^, which may have induced larger power increases in the high gamma band than a silent reading task.

Beamformer and group statistical analyses revealed that the significant ERSs in the high gamma band were localized in the left MFG (BA 46). In a previous study, activity in the left MFG reflected working memory processes related to explicit memory retrieval^30^. A repetitive transcranial magnetic stimulation (rTMS) study showed that the left prefrontal cortex was engaged in verb processing and was more active when participants were retrieving verbs than nouns^31^. Thus, the high gamma ERSs in the left MFG in our study are most likely related to the verb generation process. However, neither the group analyses nor the time-frequency analyses detected high gamma ERSs in the left MFG during the silent reading task. An ECoG study comparing overt and covert (silent) word repetition revealed that high gamma activities during covert word repetition were mainly localized to the superior temporal gyrus (STG), with less activation of the MFG, although the power was small^32,33^. Therefore, even if silent reading induced high gamma activity in the left MFG, it might be too weak to be detected by MEG.

In the present study, we detected significant high gamma ERSs related to verb generation in the group statistical analyses. However, high gamma ERSs were detected with the beamformer method in eight of the fifteen participants. Thus, in this work, the sensitivity of MEG to detect language-related high gamma activity in the verb generation task was approximately fifty percent. Therefore, applying this method in a clinical examination to determine individual language dominance remains difficult. However, if the detection sensitivity of the language-related high gamma band activity could be increased, the high gamma ERSs measured by MEG might be applicable in clinical examinations to investigate individual language dominance and localization. Thus, further improvement to detect cortical high gamma activity is required. To increase the detection sensitivity, we propose three strategies. First, increasing the number and density of MEG channels may increase the probability that a sensor will be located immediately above the activated area. Second, a task that imposes higher task demand on the prefrontal activity could be used or the trial times could be increased. Third, the time window of the beamformer analysis could be specifically optimized for individual temporal profiles of cortical activity.

In conclusion, language-related high gamma band activity was successfully detected non-invasively using beamforming MEG and group statistical analyses. This approach may be useful for more precise non-invasive evaluations of language dominance and localization. However, further improvement of the detection sensitivity of the language-related high gamma band activity is required before this approach can be applied to clinical examinations.

## Materials and Methods

### Participants

Fifteen healthy, native Japanese volunteers (21–26 years old; 1 male and 14 females) participated in this study. Edinburgh Handedness Inventory tests^34^, which were performed prior to the study, revealed that all participants were right-handed. No participants had neurological or psychiatric diseases, and their visual fields were normal or corrected-to-normal. In accordance with the Declaration of Helsinki, we explained the purpose and possible consequences of this study to all participants and obtained informed consent prior to their participation in the study. The ethics committee of Osaka University Hospital approved the study protocol.

### Task

We used a verb generation task and a silent reading task. In both tasks, control stimuli and Japanese semantic words (visual stimuli) composed of three Japanese hiragana or katakana characters were displayed for 3,000 ms. Each word was one monomorphemic and monosyllabic three-mora Japanese word (e.g., : duck or : melon) selected from a standard reference of Japanese lexical properties^35,36^. Randomized pixel images of the next word to be presented were used as control stimuli. In the rest interval between each control stimulus and semantic word stimulus, a small red fixation circle was displayed in the centre of the projection screen^1,3^.

In the verb generation task, participants were instructed to silently read each presented word once immediately after the word presentation and then to recall a verb associated with that word. In the silent reading task, participants were instructed to silently read each presented word only once. In total, 100 different words were presented serially, and the same set of 100 words was used in both tasks (Supplementary Fig. S1). The order of the presented words was random for both tasks and for the participants. The task that was presented first (i.e., the verb generation task or the silent reading task) was also randomized among participants.

### Measurements

A 160-channel, whole-head MEG system equipped with coaxial-type gradiometers (MEG vision NEO; RICOH, Tokyo, Japan) was used in a magnetically shielded room. The participants lay in a supine position on the bed with their head centred. Visual word stimuli were displayed on a projection screen 325 mm from the participant’s eye using a visual presentation system (Presentation; Neurobehavioral Systems, Berkeley, CA, USA); a liquid-crystal projector (LVP-HC6800; Mitsubishi Electric Corporation, Tokyo, Japan) was used for the display.

Anatomical magnetic resonance imaging (MRI) data were obtained using a 3.0-T magnetic resonance scanner with an 8-channel whole-head coil (Signa Excite HDxt 3.0 T; GE Healthcare, Chicago, IL, USA). The three-dimensional facial surface of each participant was scanned (FastSCAN Cobra; aranz medical, Christchurch, New Zealand) to associate the MEG data with individual MRI data. During MEG recording, five head marker coils were attached to the scalp to adjust the position and orientation of the MEG sensors on the head. The three-dimensional facial surface data were superimposed onto the anatomical facial surface calculated from the MRI data.

MEG signals were digitally registered online with a low-pass filter of 200 Hz at a sampling rate of 1,000 Hz. A notch filter at 60 Hz was used to eliminate the AC line noise. Participants were asked not to move their bodies and to watch the centre of the display without moving their eyes to reduce the noise related to muscle activity^3,10^.

### Beamformer and group statistical analyses

The MEG data were analysed with a beamformer method, which is a narrow-band adaptive spatial filtering method^17^. In spatial filtering analysis, the neural signal distribution of interest in the brain is constructed by the weighted sum of the signals recorded by the MEG SQUID sensors as the brain local rhythm changes. The weighted sum enables obtaining an unattenuated signal output from a contributing area; thus, the signal from a region of interest may be displayed alone, while that from other locations is suppressed^37^. Differential estimates of the source power of the control period and the period of interest (POI) for a selected frequency band and time window were computed as pseudo-T values^1^. The distribution of pseudo-T values was superimposed on the individual anatomical MRIs co-registered with the MEG data. Positive and negative values indicated ERSs and ERDs, respectively. ERSs and ERDs are the oscillatory changes of a certain frequency band and reflect the neural activities corresponding to a certain function^38,39^. For individual study participants, ERSs in the high gamma band were calculated as having positive pseudo-T values. We checked whether local maximum values were observed in the left MFG for each participant. We then superimposed the pseudo-T values larger than 0.1 onto the standard MNI brain.

The beamformer analyses created a volume that covered the whole brain of a participant with a voxel size of 5 × 5 × 5 mm. Significant differences between the active state and the control state were calculated in the following frequency bands: alpha (8–13 Hz), beta (13–25 Hz), low gamma (25–50 Hz), and high gamma (50–100 Hz). For all frequency bands, the control period was set from 200 to 0 ms before each stimulus onset, and the active state was defined as a time window of 200 ms after each stimulus onset. For the high gamma band, the start time of the active time window was consecutively moved with a step size of 50 ms from 0 ms to 800 ms after the stimulus onset, with an overlap of 150 ms; thus, 17 active states were created for each stimulus (Supplementary Fig. S2). For other frequency bands, the start of the active time window was consecutively moved with a step size of 200 ms from 0 ms to 1,000 ms after the stimulus onset without overlap; thus, 6 active states were created for each stimulus.

Group statistical analyses were generated to exclude brain anatomical differences between participants; thus, the common brain areas activated by the tasks could be determined. The statistical significance of ERSs/ERDs across participants was tested with Statistical Parametric mapping 8 (SPM8; Wellcome Trust Centre for Neuroimaging, London, UK). The functional images of each participant were normalized using an MRI-T1 template in SPM8. Each participant’s anatomical MRI was resliced to the same orientation and positioned to match the standard coordinates of the standard MNI brain. Voxel-level analyses were performed with a permutation test (1,024 permutations) using Statistical Non-parametric Mapping 5 (SnPM 5; Andrew Holmes and Thomas Nichols, Coventry, UK) after smoothing the variance with a 20-mm Gaussian kernel. These group statistical maps were subjected to a threshold of *p* < 0.05 (corrected) and superimposed onto the template brain using MRIcron software (University of Nottingham school of Psychology, Nottingham, UK). The BA where the statistical value of a voxel was significant was determined by converting the MNI coordinates to Talairach coordinates^40^. Results that appeared outside of the cortex were excluded. We applied a Bonferroni correction for multiple comparisons and used a statistical threshold level of corrected *p* < 0.05.

### Virtual sensors and time-frequency analyses

After estimating the tasks’ spatiotemporal peaks of ERSs and ERDs using group statistical analyses, an individual’s neuromagnetic signals corresponding to the peaks of the group statistical maps were generated by a virtual sensor. Signals from the targeted voxel were exclusively projected by the virtual sensor because residual noise was removed and signals from other parts of the brain or the extracranial environment were suppressed^3^. The oscillatory changes at the locations of the peaks found in the group analyses were shown by applying time-frequency analyses to these signals in each participant. These analyses were performed using the FieldTrip software (http://www.fieldtriptoolbox.org). The baseline ranged from −200 to 0 ms before stimulus onset, and the POI ranged from 0 to 1,500 ms after stimulus onset. The frequency range from 2 to 102 Hz was divided every 2 Hz, and spectral amplitudes were calculated in 4-Hz bins. The magnitudes of the high gamma band at 650 ms and 950 ms during each task were extracted for each participant, and a direct comparison between the verb generation task and the silent reading task was performed by a paired t-test (two-tail).

### Data availability

All data generated or analysed in this study are available from the corresponding author on reasonable request, after additional ethical approvals regarding data provision to individual institutions.

## Electronic supplementary material

Supplementary Information
