# Supplementary material for: Non-invasive detection of language-related prefrontal high gamma band activity with beamforming MEG
Source: Sci Rep. 2017 Oct 27;7:14262. doi: 10.1038/s41598-017-14452-3 (PMC5660237; doi:10.1038/s41598-017-14452-3)
Supplement: Supplementary file 1 — Supplementary Information [file 41598_2017_14452_MOESM1_ESM.pdf]

Supplementary Information for

Non-invasive detection of language-related prefrontal high gamma band activity with beamforming MEG

Hiroaki Hashimoto[1,2], Yuka Hasegawa[3], Toshihiko Araki[4], Hisato Sugata[5], Takufumi Yanagisawa[1,2,6], Shiro Yorihuji[3], and Masayuki Hirata[1,2,6]

[1] Osaka University, Endowed Research Department of Clinical Neuroengineering, Global Center for Medical Engineering and Informatics, Suita 565-0871, Japan

[2] Osaka University Graduate School of Medicine, Department of Neurosurgery, Suita 565-0871, Japan

[3] Osaka University Graduate School of Medicine, Division of Functional Diagnostic Science, Suita 565-0871, Japan

[4] Osaka University Hospital, Department of Medical Technology, Suita 565-0871, Japan

[5] Oita University, Faculty of Welfare and Health Science, Yufu 879-5503, Japan

[6] National Institute of Information and Communications Technology, and Osaka University, Center for Information and Neural Networks (CiNet), Suita 565-0871, Japan

Contents:

Supplementary Figures 1-2

Supplementary Figures

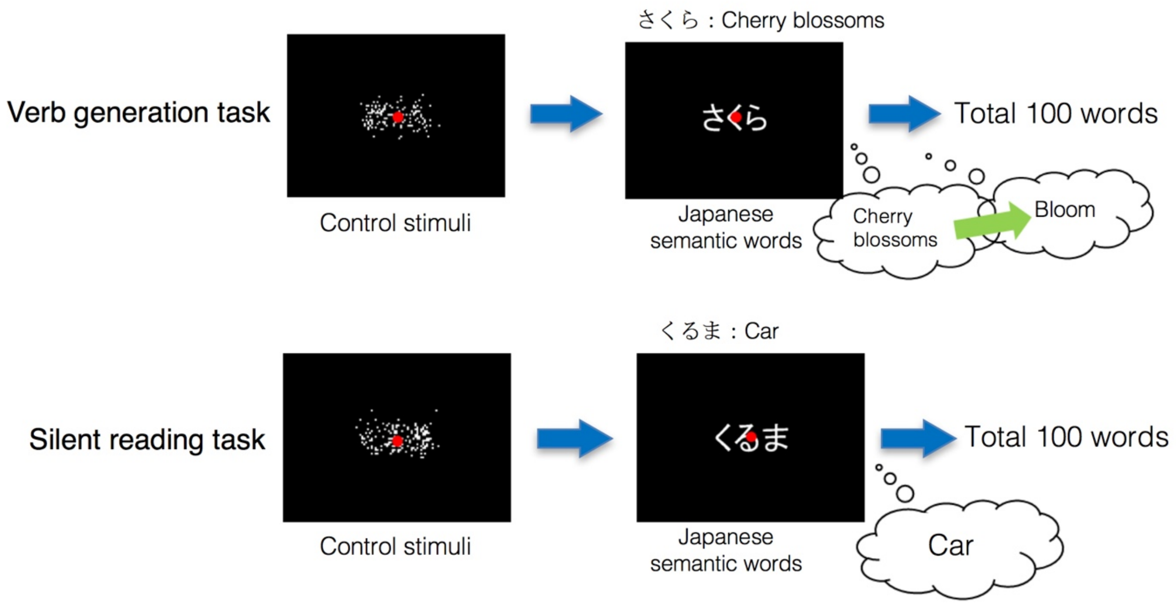

Supplementary Figure S1. A schematic diagram of the verb generation and silent reading tasks. In both tasks, 100 Japanese semantic words composed of three Japanese hiragana or katakana characters were displayed for 3000 ms after randomized pixel pictures were displayed for 3000 ms as control stimuli. In the verb generation task, subjects were instructed to silently read the presented word once immediately after its presentation and then to recall a verb associated with the word. In the silent reading task, subjects were instructed to silently read each presented word once. A total of 100 different words were presented serially, and the same set of 100 words was used in both tasks. The order of the presented words was random among both tasks and subjects.

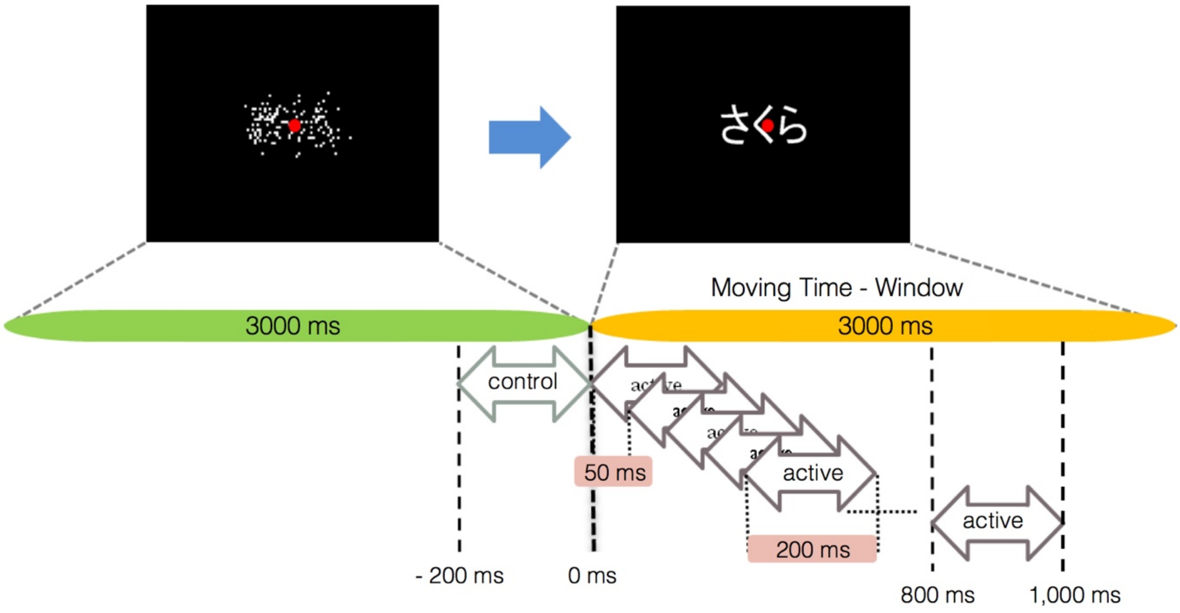

Supplementary Figure S2. A schematic diagram of the analysis used in the present study. In both the silent reading and verb generation tasks, the control period was defined as 200 ms preceding the stimulus onset (-200 to 0 ms), and the active states were defined as consecutive 200-ms windows starting at 0 ms (0 to 200, 50 to 250, 100 to 300, …, 800 to 1000 ms) with an overlap of 150 ms for the high gamma bands. For other frequency bands, the beginning time of the active time window was consecutively moved at steps of 200 ms from 0 ms to 1000 ms after stimulus onset without an overlap.
